# Dependence of mechanical properties on the phase composition of intercritically annealed medium-Mn steel as the main competitor of high-strength DP steels

**DOI:** 10.1038/s41598-024-60295-0

**Published:** 2024-04-26

**Authors:** Adam Skowronek, Adam Grajcar, Roumen H. Petrov

**Affiliations:** 1 Materials Research Laboratory, Faculty of Mechanical Engineering, Silesian University of Technology, Konarskiego 18a St., 44-100 Gliwice, Poland; 2 Department of Engineering Materials and Biomaterials, Faculty of Mechanical Engineering, Silesian University of Technology, Konarskiego 18a St., 44-100 Gliwice, Poland; 3 Research Group Materials Science and Technology, Department of Electromechanical, Systems and Metal Engineering, Tech Lane Ghent Science Park-Campus A, Ghent University, Zwijnaarde, Technologiepark 46, 9052 Ghent, Belgium; 4 Department of Materials Science and Engineering, 3mE-TUDelft, Delft University of Technology, Building 34, Mekelweg 2, Delft, 2628 CD The Netherlands

**Keywords:** Medium-Mn steel, Intercritical annealing, Hot-rolled sheet, Mechanical properties, Retained austenite, Fresh martensite, Materials for devices, Structural materials

## Abstract

Depending on the alloy composition, intercritical annealing may provide different phases in the microstructure. For low-alloyed dual-phase (DP) steels it is usually ferrite and martensite, while for medium-Mn steels retained austenite is also formed. In a present study, a wide intercritical temperature range was applied to a 5% Mn steel to investigate possible microstructure combinations: ranging from fully ferritic, through ferritic-austenitic, multiphase, to fully martensitic, which were next investigated in terms of mechanical properties to clarify the behavior of this type of material. The obtained results together with technological issues and economic indicators were next compared to mechanical properties of typical DP steels in order to assess the possibility of replacing this material in car production. The mechanical properties were evaluated using static tensile and hardness tests. The phase composition was determined qualitatively and quantitatively using dilatometry, X-ray diffraction measurements, and electron backscatter diffraction analysis. The results suggest that both initial austenite and martensite fractions have a decisive influence on the yielding and elongation of steel; however, the tensile strength depends mainly on the sum of martensite initially present in the microstructure and the strain-induced martensite formed from the plastically deformed austenite regardless of the initial retained austenite—martensite ratio. The results indicate superior total elongation of medium-Mn steels reaching 30% compared to DP steels with a similar strength level in the range between 900 and 1400 MPa. However, medium-Mn steels could be a significant competitor to dual phase steels only if some technological problems like discontinuous yielding and serrations are significantly reduced.

## Introduction

The intercritical annealing (IA)^1^ is the least complicated heat treatment of medium-Mn steels providing the formation of retained austenite (RA) among all current methods^2^. It consists of single annealing, which may be conducted both by batch^3^ or continuous annealing lines ^4^ and provides usually high fractions of RA in the ferritic matrix (F). Other methods like austempering (matrix of bainitic ferrite (B)) or quenching and partitioning (matrix of tempered martensite) beside basic furnace require additional equipment as isothermal holding during cooling or even an increase of the temperature after partial quenching for partitioning have to be performed^5^. Moreover, intercritical annealing allows for relatively simple control of the wide range of produced phase composition because time and temperature of this process are the most important parameters. However, it should be taken into account that a alight change in temperature or time significantly affects the properties obtained due to the relatively narrow technological window^6,7^. Steels containing retained austenite also have disadvantages compared to conventional steels. One of them is poorer machinability resulting from the dynamic martensitic transformation during deformation due to cutting of the material. The martensite formed in the cutting area is a hard and brittle phase that may result in poor surface finish, poor machinability and high tool wear^8^.

The research on intercritical annealing of medium-Mn steels has shown that it is possible to obtain usually between 25 and 50% of RA in steel, depending on its chemical composition and selected process parameters^1^. It results frequently in ultimate tensile strength (UTS) of over 700 MPa and total elongation (TEl) of over 25%^2^. This suggests that medium-Mn steels may be a direct competitor to dual phase (DP) steels because the strength properties that can be obtained are at a similar level ^9^, while plasticity that can be achieved in case of medium-Mn steels may be significantly improved^10^. The significant advantage in mechanical properties of medium-Mn steel can compensate for the slightly worse economic indicator of this material compared to DP steel. The DP steels are usually low-alloy steels^11^, which translates into low production costs. In the case of medium-Mn steel, it is necessary to enrich the alloy with manganese in the range of 3–12% as well as other elements such as Si and Al, which increases the cost of the alloy^1,4,12^. The heat treatment process itself is very similar in both cases because it consists of intercritical annealing, which, however, results in a different final phase composition. Before industrial implementation of medium-Mn steels, problems such as the occurrence of discontinuous yielding^13^ or the effect of non-uniform deformation (DSA effect)^13^ must also be solved.

In case of DP steels the final mechanical properties are determined by annealing temperature because it controls a fraction of high temperature austenite, and therefore the final martensite fraction in the microstructure^14^. In the case of medium-Mn steels, scientists' efforts are mainly focused on obtaining the highest possible RA fraction to achieve high plasticity. The tests of mechanical properties are mainly devoted only to samples exhibiting stability of RA at room temperature^6,15–18^. This makes it impossible to characterize changes in mechanical properties for the entire intercritical range, and thus makes it difficult to directly compare the properties of medium-Mn steels and DP steels. For this reason, the aim of the current work is to characterize the evolution of mechanical properties of Al-alloyed medium-Mn steel with the change of its phase composition (tempered martensite, ferrite, retained austenite and fresh martensite) controlled by a wide range of intercritical annealing temperatures. Moreover, the article contains a critical comparison of medium-Mn steels and DP steels in terms of production process, economic indicators, mechanical properties, being the novelty and the added value of this paper to a common knowledge on AHSS steels, which is very important for the further development of the automotive industry.

## Methods

**Figure 1:**
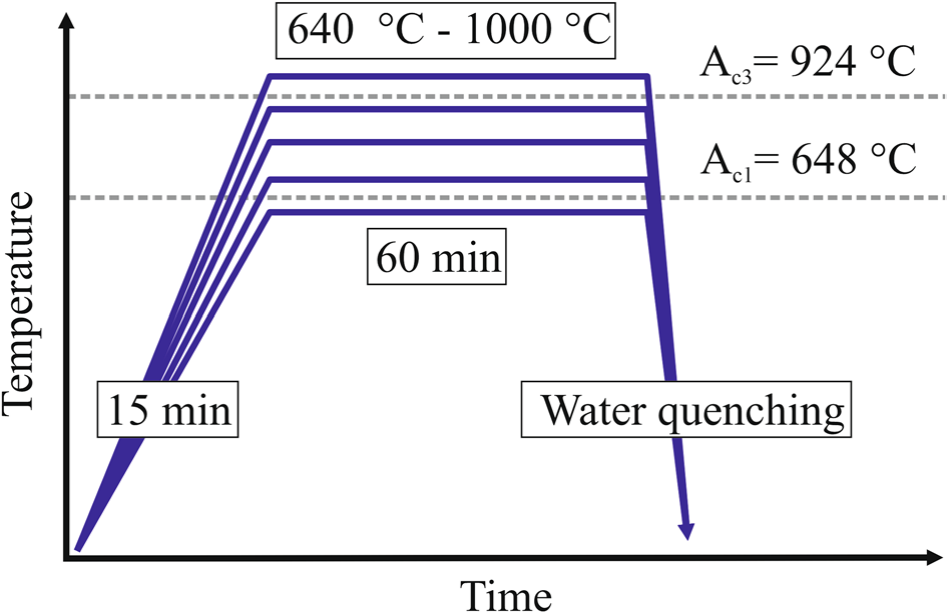
Heat cycle schedule used during the investigations.

The chemical composition of the investigated steel is presented in Table 1. It is a 4.5 mm thick hot-rolled plate with the martensitic microstructure. The further details regarding melting, casting and hot working of the steel may be found in our earlier papers^10,19^. According to our previous research the addition of 1.7% of Al results in a intercritical range from 648 °C (A_C1_) to 924 °C (A_C3_) enabling wide tailoring of possible phase fractions and composition^19^. Due to the addition of 0.16% C and 4.7% Mn the intercritical region allows for obtaining a high proportion of intercritical austenite up to about 40%^10^. In medium-Mn steels, both Mn and C are intended to chemically stabilize the retained austenite, but to improve weldability of the steel, a carbon concentration must be limited. Therefore, stabilization is achieved to a greater extent by manganese. However, despite the relatively low carbon concentration, it is still an important austenite stabilizer due to its redistribution to the intercritical austenite^10^. The present study is devoted to characterization of mechanical properties for different phase compositions of the alloy after the intercritical annealing for 60 min at temperatures 640, 660, 680, 700, 720, 760, 800 and 1000 °C (Fig. 1), which covers the entire intercritical range.

**Table 1:** Chemical composition of the hot rolled steel plate used for the investigations.

Chemical element, wt.%					
C	Mn	Al	Si	Mo	Fe-balance
0.16	4.7	1.6	0.20	0.20	93.12

**Figure 2:**
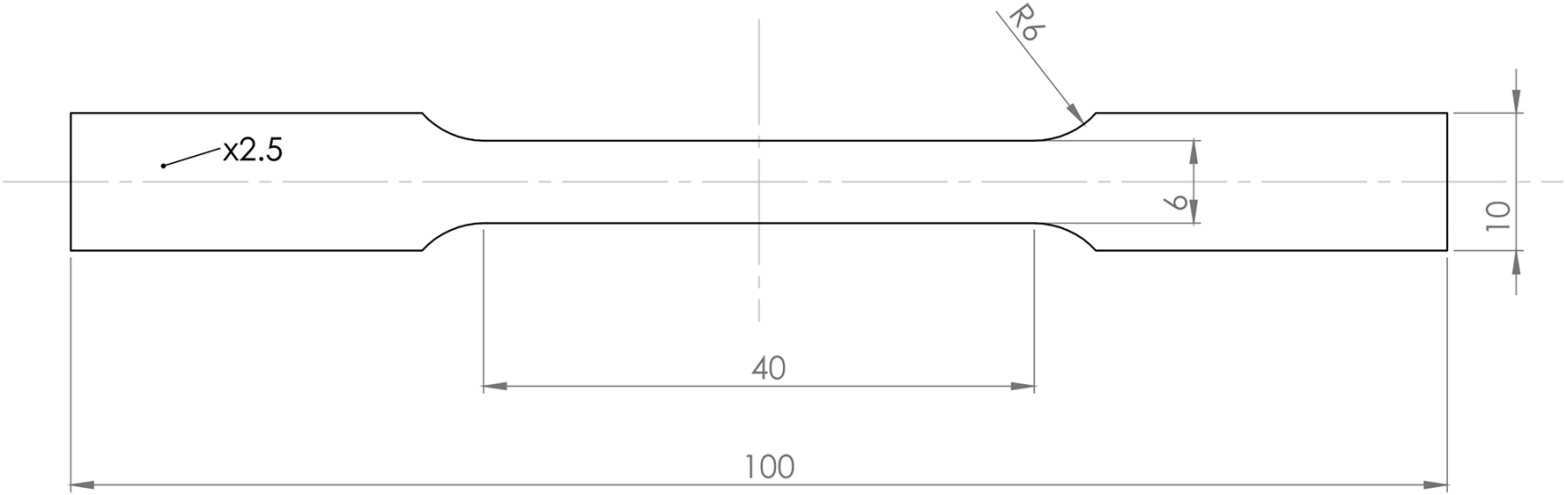
The geometry of used tensile samples.

The martensite-start temperature (M_s_) of austenite after annealing at different temperatures was determined by dilatometry on ⌀4 × 10 mm samples using Bahr 805A/D dilatometer, equipped with fused silica push-rods and a S-type thermocouple. The tensile samples (Fig. 2) with a gauge length of 40 mm, 6 mm wide and 2.5 mm thick (according to ASTM E8 standard)^20^ were heat-treated using Nabertherm LT15/12/P330 muffle furnace under argon atmosphere. The samples were heated for 15 min to reach the set temperature, annealed for 60 min at a constant temperature and finally water cooled to room temperature (Fig. 1). The heating time needed to reach the annealing temperature was determined by measuring the heating rate of the core of 2.5 mm thick plate using PCE-T390 datalogger and K-type thermocouple.

Both dilatometric and tensile samples were cut and machined from the initial plate along the rolling direction. The static tensile test was performed according to ASTM E8 standard^20^ at a nominal strain rate of 10^–3^ s^−1^ using ZWICK 020 universal testing machine equipped with an extensometer. The instantaneous strain hardening exponent within the uniform deformation range has been calculated using the following equation ^21^:

1 $$n=\frac{{\varepsilon }_{T}d{\sigma }_{T}}{{\sigma }_{T}d{\varepsilon }_{T}}=\frac{d(log{\sigma }_{T})}{d(log{\varepsilon }_{T})}$$

where: *n* is the strain hardening exponent, ${\sigma }_{T}$ is the true stress and ${\varepsilon }_{T}$ is the true strain.

Hardness measurements were conducted using a FB-700 Vickers tester at a load of 1 kgf. Hardness was measured for heat treated and deformed samples in the UEl and TEl areas (in the neck, 1 mm from the fracture point). Ten measurements for each sample were taken and the mean hardness determined after discarding the two extreme values.

The ferrite and high temperature austenite proportions were calculated in each sample using a method proposed in work^19^ basing on changes in linear expansion ratio determined using dilatometry. Retained austenite fraction was calculated using X-ray diffraction performed on Panalytical X’Pert Pro MPD diffractometer. The device was equipped with the cobalt anode (λK_α_ = 0.179 nm) and the PIXcel 3D-detector. The diffraction lines were recorded in the Bragg–Brentano geometry in the angular range of 45–105° (2θ), with the step of 0.026° and a scan speed of 0.6 s/step. The RA fraction was determined using High Score Plus software (Version 3.0) and Rietveld refinement method. The fraction of fresh martensite (MF) in each sample was calculated by subtracting the RA fraction (XRD) from the calculated value of high-temperature austenite (dilatometry).

The EBSD technique was used to obtain more detailed information about the microstructure. The EBSD system was mounted on FEI Quanta FEG 450 SEM with field emission gun (FEG) filament. The EBSD was performed at 20 kV on the sample tilted 70° and a probe current of ~ 2.5 nA using a hexagonal scan grid with 60 nm spacing. The EBSD data was processed using the OIM-TSL data analysis software v.7.3.1. A double cleaning procedure was applied on the original orientation data. First, a grain confidence index (CI) standardization was used employing a grain tolerance angle of 5° and a minimum grain size greater than 3 pixels^22^. Next, the neighbor orientation correlation with CI = 0.1 and clean up level 3 was used. The points with CI ≤ 0.1 were removed from the analysis as doubtful. The image quality (IQ) and phase (P) maps were applied to characterize the microstructure. The dark regions in IQ maps of alpha phase, which contain higher density of lattice imperfections^23^, were assigned to martensite^24,25^. The method based on differentiating the range of grain average IQ (GAIQ) values for ferrite and martensite^26,27^ was used to distinguish these phases and calculate their surface fractions. The strain distribution in analyzed samples was investigated using the kernel average misorientation (KAM) values^28,29^, which provides quantitative information on the misorientation between the neighboring pixels (concerning 3rd nearest neighbor and 5° upper cut off limit ) acquired via EBSD.

This work is devoted to determining the relationship between the mechanical properties of the alloy and the summary characterization of its microstructural state. An extensive analysis focused on detailed microstructural features, i.e. austenite formation kinetics, evolution of chemical composition of austenite, martensite tempering, the influence of the size, morphology and chemical composition of retained austenite on its chemical and mechanical stability, is available in our previous works^10,19^.

## Results

**Figure 3:**
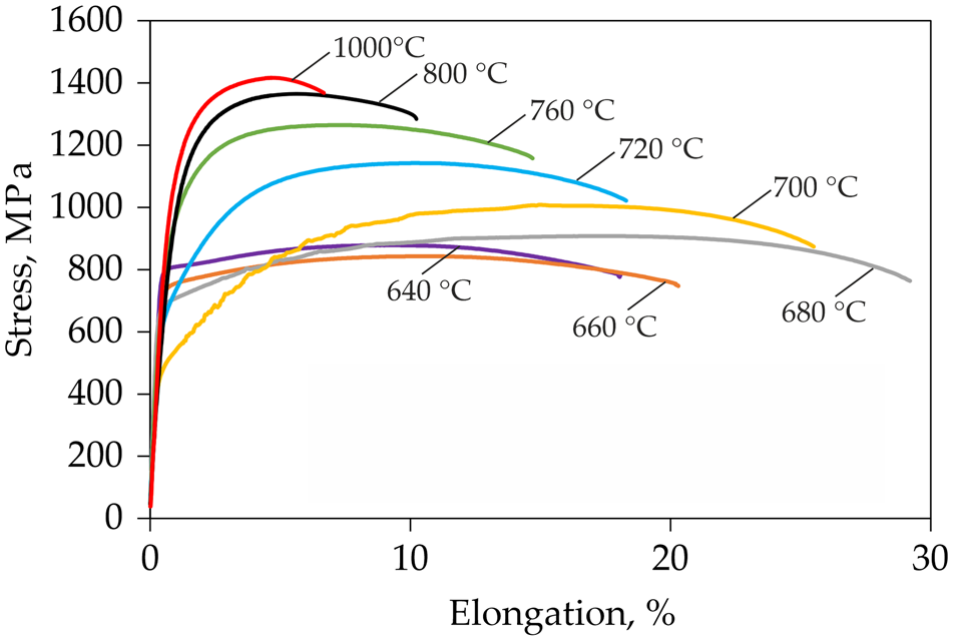
The engineering stress—elongation tensile curves for samples annealed for 1 h at different temperatures.

**Figure 4:**
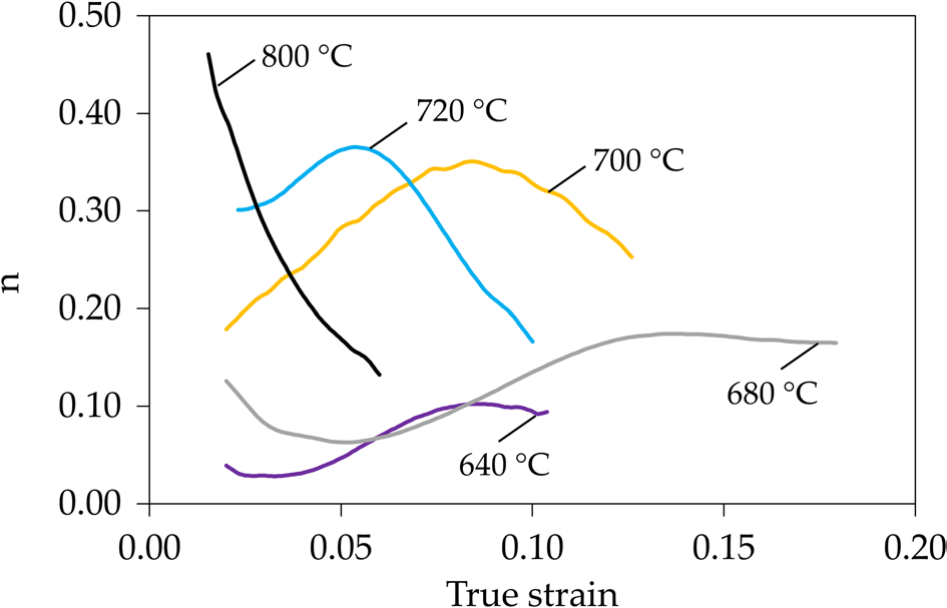
Instantaneous strain hardening exponent—true strain curves for selected samples annealed at different temperatures for 1 h.

The tensile curves of samples annealed at different temperatures and thus characterized by different microstructure are presented in Fig. 3. Samples annealed at 640 and 660 °C are characterized by a relatively high yield strength (YS) reaching respectively 803 and 740 MPa. After yielding the stress increase related to strain hardening (SH) is negligible as it reaches only about 75 and 100 MPa for the samples annealed at 640 and 660 °C, respectively. The strain hardening exponent (*n*) for sample annealed at 640 °C reaches a value of only about 0.11 (Fig. 4), which results in elongation limited to 18%. The sample annealed at 680 °C exhibits the highest elongation among all samples of about 30% (Fig. 3). It is related to the continuously increasing strain hardening exponent reaching up to 0.2 (Fig. 4). Such behavior is characteristic for microstructures containing over 30% of retained austenite^30,31^. YS of the sample annealed at 680 °C is lower than these of samples annealed at lower temperatures reaching 670 MPa; however, the UTS is higher as it reaches 910 MPa. The sample annealed at 700 °C exhibits the lowest YS among all samples of about 530 MPa. However, the further high strain hardening allows to obtain UTS of 1000 MPa (Fig. 3). Strain hardening for this sample is at a very high level because its exponent reaches a value of about 0.35 at a true strain of 0.8 (Fig. 4). However, at higher deformations the strain hardening of the sample annealed at 700 °C decreases rapidly—differently to the sample annealed at 680 °C—which provides in the limited elongation of 25.5%. The sample annealed at 700 °C also shows non-uniform deformation visible as serrations in the tensile curve, but this topic will be discussed later. With the further increase of intercritical annealing temperature up to 1000 °C a significant decrease in sample’s elongation to 8.5% was measured (Fig. 3), which is accompanied with a rapid increase in YS (up to 1080 MPa) and UTS (up to 1420 MPa) values. For a sample annealed at 720 °C, the strain hardening exponent increases to a strain of 0.5 and then decreases. However, in the case of samples annealed at higher temperatures, the strain hardening exponent shows a continuous and rapid decrease (Fig. 4), which is characteristic for high strength DP and TRIP steels^32,33^.

**Figure 5:**
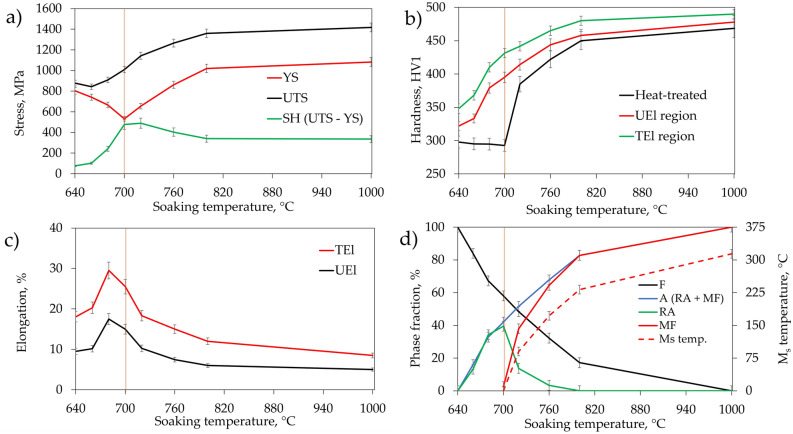
Summary data for the evolution of mechanical properties and microstructural state with an increase of intercritical annealing temperature: the evolution of (**a**) strength properties, (**b**) hardness, (**c**) plastic properties, (**d**) phase composition and stability of retained austenite. *YS* yield strength, *UTS* ultimate tensile strength, *SH* strain hardening expressed by the increase in stress between YS and UTS values, *TEl* total elongation, *UEl* uniform elongation, *F* ferrite, *A* austenite, *RA* retained austenite, *MF* fresh martensite, *M*_*s*_* temp.* martensite start temperature. The detailed data regarding the determination of the phase composition of steel and RA stability are available in our previous work^19^.

With the initial increase of the intercritical annealing temperature up to 700 °C the softening of the initial martensitic microstructure^34^ is visible because YS (Fig. 5a) and hardness (Fig. 5b) of the alloy decrease, whereas elongation values (Fig. 5c) increase. These changes are also influenced by the formation of retained austenite during heat treatment, which is shown in Fig. 5d. The formation of retained austenite affects especially UEl and TEl because strain-induced transformation of retained austenite to martensite (SIMT) causes a strong increase of strain hardening and thus delays the formation of the neck (TRIP effect). However, the sample annealed at 700 °C despite having the highest fraction of RA (40%) shows limited elongation in comparison to the sample annealed at 680 °C. It is caused by relatively low stability of retained austenite which is reflected by M_s_ temperature changes plotted in Fig. 5d. The changes in the M_s_ temperature indicate that the stability of austenite decreases rapidly with increasing soaking temperature. The M_s_ temperature for samples soaked below 700 °C was not recorded during dilatometric tests. For soaking temperatures higher than 700 °C, the M_s_ temperature is recorded by dilatometry and increases up to 314 °C after soaking at 1000 °C. The decrease in RA stability of the sample soaked at 700 °C in comparison to the sample soaked at 680 °C results in very high strain hardening in the first stage of deformation (Figs. 3 and 4) but the TRIP effect is quickly exhausted, causing simultaneously a rapid increase in the amount of fresh martensite in the microstructure and thus a decrease in both uniform and total elongations. This results show that not only a fraction of retained austenite but also its stability is crucial in terms of mechanical properties. The further increase in annealing temperature results in a reduction of ferrite fraction in samples from ~ 60% at 700 °C to 17 and 0% at 800 °C and 1000 °C, respectively, and formation of fresh martensite from low-stable austenite (M_s_ increase) during final cooling from ~ 1% after annealing at 700 °C to 83% and 100% after annealing at 800 and 1000 °C, respectively. The microstructure hardening caused by changes in phase composition results in an increase of YS between samples annealed at 700 and 1000 °C by 550 MPa and reduction of TEl by 17%.

Regarding the UTS values, the initial microstructure generated during heat treatment seems to have limited influence. The UTS values increase continuously with the increase of annealing temperature from 880 MPa after annealing at 640 °C up to 1420 MPa after annealing at 1000 °C, regardless of the RA and MF ratio. Therefore, both the presence and stability of RA do not have a significant influence on UTS of intercritically annealed medium-Mn steel as it was in case of YS, despite the highest SH of 490 MPa was recorded for sample annealed at 700 °C containing the highest RA fraction of 40%. The UTS value is rather dependent on a final martensite fraction (formed during cooling and sample deformation) in the microstructure. This relation has not been yet pointed in the literature for medium-Mn steels due to the frequent analysis and comparison of results only for samples characterized by thermal stability of austenite at room temperature^3,6^. A similar tendency occurs in results of hardness measurements performed on deformed samples in UEl and TEl (neck) areas. Hardness increases continuously from 320 HV1 (for uniform elongation region) of sample annealed at 640 °C to 480 HV1 for sample annealed at 1000 °C, with an increase of cumulated martensite fractions formed during cooling and deformation. Hardness in the neck area is usually about 20 HV1 higher than in the UEl area. The changes in ferrite fraction do not contribute clearly to the UTS and hardness values of the alloy.

**Figure 6:**
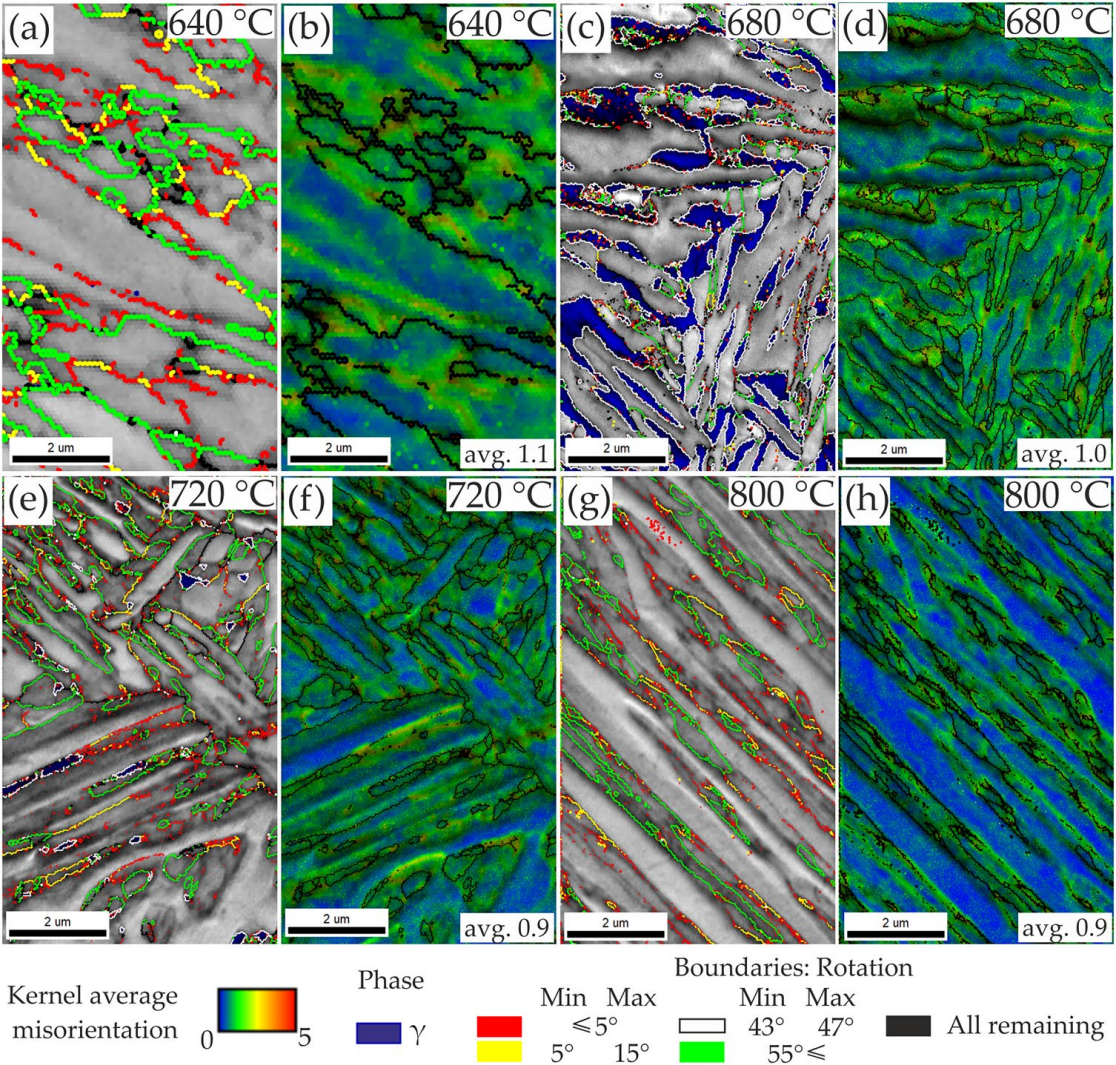
Image quality (IQ) with a superimposed phase maps (**a,c,e,g**) with indicated austenite phase (dark blue) and grain boundary misorientations and Kernel Average Misorientation (KAM) maps (**b,d,f,h**)for samples annealed at different temperatures for 1 h.

The microstructural details were investigated by EBSD analysis in Fig. 6. The maps indicating grain misorientation values show that samples contain three main types of grain boundaries. Sample annealed at 640 °C (Fig. 6a) contains mainly low angle (< 15°) and high angle (> 55°) boundaries. This are the characteristic grain misorientations for martensitic, tempered martensitic and bainitic structures^35,36^. In sample annealed at 680 °C (Fig. 6c) the grain boundaries with misorientations of ~ 45° dominate. They represent the K-S and N-W grain boundary relationships between ferrite and austenite^37^. The high proportion of this type of grain boundaries indicates high homogeneity of the microstructure and alternating arrangement of ferrite and retained austenite. For samples annealed at temperatures higher than 700 °C—where martensitic transformation occurred during cooling—the martensite-related grain boundaries misorientations reappear (Fig. 6e,g). The average KAM values representing strain distribution decrease with an increase in annealing temperature from 1.1° for sample annealed at 640 °C to 0.8° for sample annealed at 1000 °C indicating samples softening. The microstructure relaxation occurs especially in the ferrite matrix, where an average KAM value decreases from 1.1° (640 °C) to 0.43° (1000 °C) (Table 2). In case of retained austenite or fresh martensite their strain is much higher and even increases in comparison to the initial microstructure as the average KAM value reaches up to ~ 1.6° for sample annealed at 800 °C, respectively.

**Table 2:** Influence of intercritical annealing temperature on kernel average misorientation in selected samples.

Annealing temperature, °C	Average KAM, ° ± 0.03		
	All	F matrix	RA + MF
640	1.10	1.1	–
680	1.01	0.9	1.22
720	0.89	0.56	1.35
800	0.78	0.43	1.57

**Figure 7:**
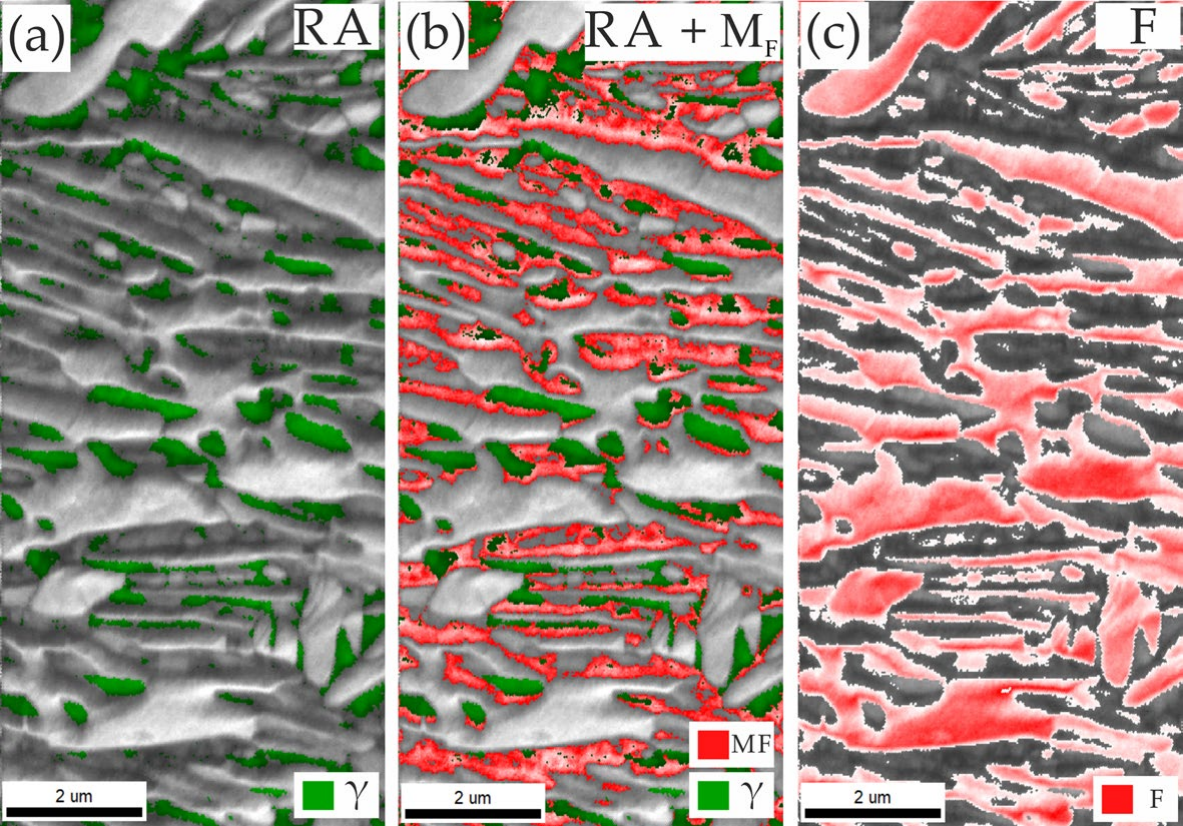
Image quality (IQ) maps with indicated (**a**) RA, (**b**) RA + MF and (**c**) F regions determined on the basis on IQ values in the sample annealed at 700 °C.

The IQ + phase maps presented in Fig. 7 clearly show that the martensitic transformation took place in the sample annealed at 700 °C. The sum of measured RA and MF fractions are equal to ~ 41%, which is in agreement with the RA fraction measured by XRD method (Fig. 5d), which suggests that the austenite was transformed during the sample preparation process due to its very low stability. The difference in measurements by XRD and EBSD methods may also result from the limitations of the latter. The EBSD method is incapable of resolving austenite films with dimensions smaller than 50–70 nm^38,39^, which may be found in the analyzed structures.

The more detailed characterization of microstructure and chemical stability of retained austenite in the investigated steel may be found in our previous paper^19^.

## Discussion

Automotive sheet steels are subject to a number of requirements regarding: strength, toughness, ductility, and durability; fatigue resistance; weight reduction; cost-effectiveness, formability and processability, crash performance. Specific levels of a given property depend on the area of use and overall part’s responsibility, geometry, vehicle type and even producer. On the one hand, elements used in the crumple zone must be characterized by moderate tensile strength (> 600 MPa), high plasticity (> 20%), high strain hardening and the ability to absorb energy as they should provide controlled deformation. On the other hand, elements of the passenger compartment must be characterized by the highest possible tensile strength (980–1800 MPa) so as not to be deformed during crush events^40–42^.

**Figure 8:**
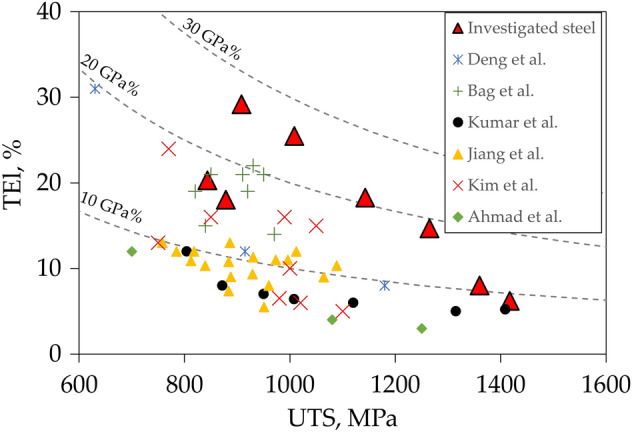
Comparison of the mechanical properties of dual-phase steel available in the literature (Deng et al.^9^, Bag et al.^14^, Kumar et al.^44^, Jiang et al.^45^, Kim et al.^46^, Ahmad et al.^47^) and the investigated medium-Mn steel.

The obtained mechanical properties of medium-Mn steel samples intercritically annealed at different temperatures have been plotted in Fig. 8 together with results of mechanical properties of different DP steels available in the literature. It is visible that dual-phase steels, despite different plasticity and strength levels, do most often not exceed the UTS × TEl product threshold of 20 GPa%^43^. The intercritical annealing of analyzed medium-Mn steel allowed to obtain UTS × TEl over 20 GPa% for four samples annealed in a temperature range from 680 to 760 °C. It reached maximum of ~ 26 GPa% for the sample annealed at 680 °C for 1 h due to over 34% of stable RA. Moreover, significantly improved elongation (TEl up to about 30%) in comparison to typical DP steels (TEl up to about 22%) exhibiting a similar strength level was obtained for medium-Mn samples annealed at the temperatures between 680 and 1000 °C. Only two samples of medium-Mn steel annealed at the lowest temperatures of 640 and 660 °C show lower elongation. However, for these samples the annealing took place at a temperature below or only slightly above A_c1_ temperature (648 °C), which did not allow for the formation of high austenite fraction during annealing. Tests have shown that medium-Mn steels have higher plasticity than DP steels while maintaining similar strength levels. This suggests their use in elements previously made of different DP steels (crumple zone and parts of safety cage) as they meet the same requirements for mechanical properties. However, the research is focused on the basic mechanical properties of steel, such as elongation, yield strength and tensile strength. It is still necessary to specify the specific properties mentioned earlier.

**Figure 9:**
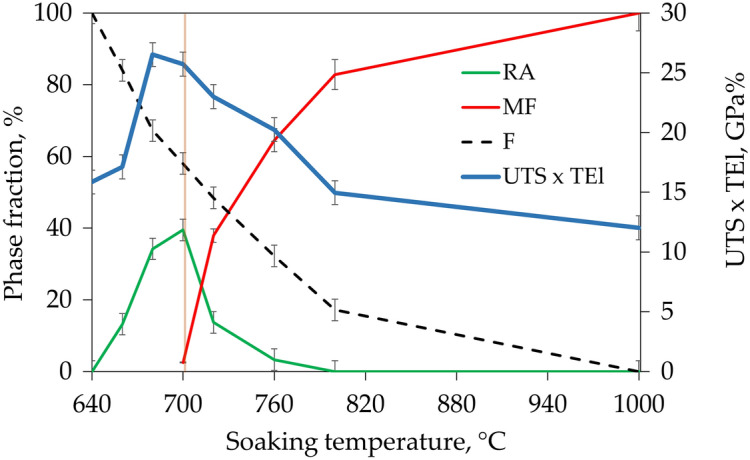
Effect of RA and MF fractions in the ferritic matrix on strength and elongation product in the investigated medium—Mn steel.

The greatest increase in elongation compared to DP steel is observed in the samples annealed at 680 and 700 °C (Fig. 8) that contain over 34% of RA in the ferritic matrix and do not have fresh martensite in the microstructure (Fig. 9). This is due to the relatively low yield point and very strong strain hardening during straining. The gradual strain-induced martensite formation delays a moment of neck formation, increasing the elongation, and at the same time allows for a significant increase in the strength of the steel relative to the YS value (SH up to 490 MPa for sample annealed at 700 °C). Samples containing initially some fraction of fresh martensite still exhibit improved elongation in comparison to DP steels; however, the elongation difference decreases because the UTS x TEl product for these samples also decreases.

The improvement of elongation and UTS × TEl product compared to DP steels is possible due to the presence of retained austenite in steels such as TRIP or medium-Mn. However, in the case of the former, the mechanical property improvement is limited as a fraction of RA in conventional TRIP steels usually does not exceed 15%^48^. Moreover, their heat treatment requires controlled multi-step cooling, which increases the production cost. In case of medium-Mn steels, their heat treatment schedule is very similar to DP steels, and they may be even air-cooled due to high hardenability, which may reduce slightly the production costs. On the other hand, the cost of medium-Mn steel is usually higher than DP steels due to increased additions of Mn and Si or Al. Table 3 shows an example cost for addition of 1 wt.% of a given alloying element to 1 tone of steel^2^. The medium-Mn alloy analyzed in the current work, according to the presented data would cost about 170 USD/ton, while a typical DP steel should not exceed about 120 USD/ton^9,14,44,46,49^. For the economic reasons the differences in price have to be balanced by the obtained properties. The value of (USD/ton)/(UTS × TEl product) for the variant of the analyzed medium-Mn steel characterized by the highest UTS × TEl product is about 6.4 USD/GPa% for 1 ton. In the case of DP steels included in Fig. 8, the value of (USD/ton)/(UTS × TEl product) for the steel with the highest UTS × TEl product (20.5 MPa%) is about 6.0 USD/GPa% for 1 ton. However, for the DP steel with an average UTS × TEl product (10.5 MPa%) it increases to over 11.4 USD/GPa% for 1 ton. It shows that the production of medium-Mn steels may be economically more justified taking into account the cost of alloy producing and the mechanical properties that can be obtained.

**Table 3:** Price of steel alloying elements^2^.

Chemical element	Fe	Mn	Al	Si	Mo	Cr	Nb	Ti	V	Ni
Cost per wt.% in 1 ton, USD	0.29	20.7	18	19	52.4	44.5	68.5	5.2	44	119

**Figure 10:**
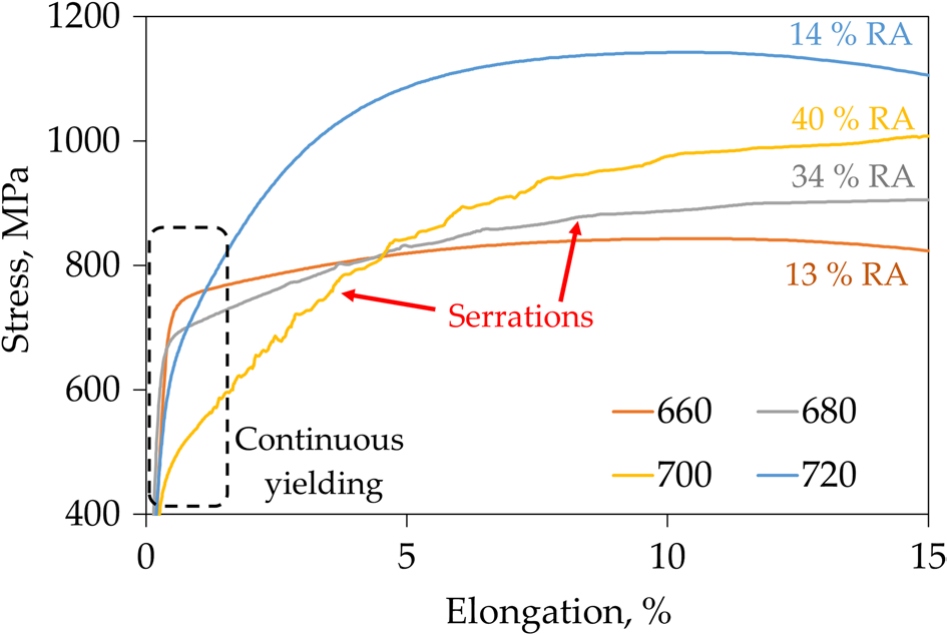
The influence of the RA fraction of the samples on the deformation behavior during uniaxial tension, illustrated in an enlarged fragment of the selected stress—elongation tensile curves.

**Figure 11:**
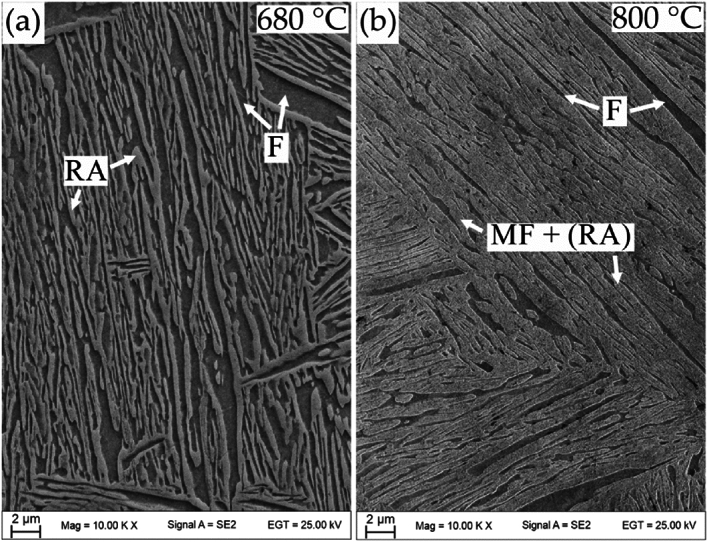
SEM micrographs of samples annealed at (**a**) 680 °C and (**b**) 800 °C showing a fine and lath-like microstructures regardless the annealing temperature.

At this point of development of medium-Mn steels also the plastic behavior problems have to be eliminated for the industrial application^1^. Cold-rolled medium-Mn steels with globular morphology exhibit discontinuous yielding^1^, which according to the literature are caused by strain partitioning between soft recrystallized ferrite grains and solution hardened retained austenite exhibiting high dislocation density^50^. The medium-Mn steel analyzed in the current work exhibits continuous yielding (Fig. 10) due to initial lath-type hot-rolled microstructure. Due to a lack of cold rolling, the matrix does not undergo recrystallization during annealing retaining high dislocation density in ferrite, which together with austenite provides continuous yielding. The annealing of hot rolled medium-Mn steel leads to obtaining a lath-like morphology present in Fig. 11 due to recrystallizationless process called Austenite Reverse Transformation (ART)^51^ regardless the annealing temperature. Unlike conventional TRIP-aided multiphase steels or bainite-based TRIP steels, there are basically no blocky grains of retained austenite in the microstructure, which significantly deteriorate ductility due to the rapid strain-induced martensitic transformation^48^. The results indicate that RA obtained in ART process exhibits increased KAM values (indication for accumulated strain). The ferrite matrix, despite softening during annealing, also retains some level of strain (Table 2). The method to eliminate the discontinuous yielding in case of cold-rolled medium-Mn steels is a former austenitization prior to intercritical annealing, which enables phase transformation without prior recrystallization^52^. However, it requires another thermal cycle increasing the production cost, which may be avoided by using hot-rolled steels.

Another important factor inhibiting the introduction of medium-Mn steels into industrial production is the instability of deformation (serrations) occurring in both cold- and hot-rolled steels, which may result in manufacturing defects such as cracks or local thinning of the sheet in stamped elements^13^. This phenomenon has been also observed in case of analyzed steel as seen in Fig. 10. It seems to be directly related to the fraction and stability of RA as it is only observed in case of samples annealed at 680 and 700 °C containing over 30% RA, and is the most intense for the sample annealed at 700 °C containing 40% of low-stable RA. For samples annealed at temperatures lower than 680 °C, where the fraction of austenite is lower than 15% and characterized by higher stability, this phenomenon does not occur. Moreover, for samples annealed at temperatures higher than 700 °C the deformation instability is not observed despite the 14% of low-stable RA (M_s_ temperature of 90 °C) fraction present in the sample annealed at 720 °C. The explanation of serrations phenomenon is an interaction between dislocations and Mn–C clusters, which may be reduced by limitation of C and Mn additions in steel^53^. The limitation of serration may be also obtained by the use of hot-rolled steel, which was showed in the present study. However, the serrations were still observed to some limited extent. The literature still does not provide a clear explanation of the cause and a effective method of eliminating this phenomenon in medium manganese steels with high fractions of RA, which is a significant obstacle to their introduction to the market^13^.

## Conclusions

The work addressed the effect of phase fractions tailored by intercritical annealing temperature on mechanical properties of 0.16C–4.7Mn–1.6Al–0.2Si–0.2Mo medium-Mn steel. The change of mechanical properties for samples containing different fractions of phases like ferrite, retained austenite and fresh martensite was explained. It was put forward that medium-Mn steels are a direct competitor to DP steels. Both steels were analyzed in detail in terms of differences in the chemical composition, alloy costs, mechanical properties and technological problems. The research led to the following conclusions:

- As the intercritical annealing temperature increases, the structure of medium-Mn steel consists of a decreasing fraction of ferrite and an increasing fraction of retained austenite, which is next gradually replaced by fresh martensite until 100% after performing annealing at 1000 °C.
- The highest fraction of RA possible to retain in the analyzed steel is 40% during 60 min annealing at 700 °C; however, the highest elongation of 30% was obtained in the sample annealed at 680 °C containing 34% RA due to its higher stability.
- The fraction of high temperature austenite controls mainly the final UTS of the steel regardless of its stability during cooling. It is reflected in continuously increasing UTS with the increase of high temperature austenite fraction, which during cooling or deformation transforms to fresh martensite.
- The elongation of analyzed medium-Mn steel is highly sensitive to the presence of fresh martensite in the microstructure and a fraction and stability of RA. Samples containing fresh martensite show a significant drop of TEl. In general, the elongation increases with increasing RA fraction.
- The analyzed medium-Mn steel exhibits significantly improved tensile elongation that translates into a higher UTS × TEl product in comparison to DP steels in a wide strength range. Therefore, medium-Mn steels may be a significant competitor for currently available DP steels despite the increased cost of the alloy. However, it is necessary to first eliminate negative issues such as discontinuous yielding and serrations during plastic deformation.

## Data Availability

The datasets used and/or analyzed during the current study are available from the corresponding author on reasonable request.
